# Supplementary material for: Neural correlates and reinstatement of recent and remote memory in children and young adults
Source: eLife. 2025 Dec 5;12:RP89908. doi: 10.7554/eLife.89908 (PMC12680376; doi:10.7554/eLife.89908)
Supplement: MDAR checklist [file elife-89908-mdarchecklist1.docx]

**Materials Design Analysis Reporting (MDAR)**

**Checklist for Authors**

The [MDAR framework](https://osf.io/xfpn4/) establishes a minimum set of requirements in transparent reporting mainly applicable to studies in the life sciences.

*eLife* asks authors to **provide detailed information within their article** to facilitate the interpretation and replication of their work. Authors can also upload supporting materials to comply with relevant reporting guidelines for health-related research (see [EQUATOR Network](http://www.equator-network.org/%20)), life science research (see the [BioSharing Information Resource](http://biosharing.org/)), or animal research (see the [ARRIVE Guidelines](http://www.plosbiology.org/article/info:doi/10.1371/journal.pbio.1000412) and the [STRANGE Framework](https://doi.org/10.1038/d41586-020-01751-5); for details, see *eLife*’s [Journal Policies](https://reviewer.elifesciences.org/author-guide/journal-policies)). Where applicable, authors should refer to any relevant reporting standards materials in this form.

For all that apply, please note **where in the article** the information is provided. Please note that we also collect information about data availability and ethics in the submission form.

**Materials:**

| **Newly created materials** | **Indicate where provided: section/figure legend** | **N/A** |
| --- | --- | --- |
| The manuscript includes a dedicated "materials availability statement" providing transparent disclosure about availability of newly created materials including details on how materials can be accessed and describing any restrictions on access. | Data availability statement/The code for the analyses presented in this paper is openly accessible at https://github.com/iryna1schommartz/memokid_fmri.  The behavioral and neuroimaging data has been deposited at https://osf.io/grku2/ and are publicly available. |  |
|  |  |  |
| **Antibodies** | **Indicate where provided: section/figure legend** | **N/A** |
| For commercial reagents, provide supplier name, catalogue number and [RRID](https://scicrunch.org/resources), if available. |  | N/A |
|  |  |  |
| **DNA and RNA sequences** | **Indicate where provided: section/figure legend** | **N/A** |
| Short novel DNA or RNA including primers, probes: Sequences should be included or deposited in a public repository. |  | N/A |
|  |  |  |
| **Cell materials** | **Indicate where provided: section/figure legend** | **N/A** |
| Cell lines: Provide species information, strain. Provide accession number in repository OR supplier name, catalog number, clone number, OR RRID. |  | N/A |
| Primary cultures: Provide species, strain, sex of origin, genetic modification status. |  | N/A |
|  |  |  |
| **Experimental animals** | **Indicate where provided: section/figure legend** | **N/A** |
| Laboratory animals or Model organisms: Provide species, strain, sex, age, genetic modification status. Provide accession number in repository OR supplier name, catalog number, clone number, OR RRID. |  | N/A |
| Animal observed in or captured from the field: Provide species, sex, and age where possible. |  | N/A |
|  |  |  |
| **Plants and microbes** | **Indicate where provided: section/figure legend** | **N/A** |
| Plants: provide species and strain, ecotype and cultivar where relevant, unique accession number if available, and source (including location for collected wild specimens). |  | N/A |
| Microbes: provide species and strain, unique accession number if available, and source. |  | N/A |
|  |  |  |
| **Human research participants** | **Indicate where provided: section/figure legend) or state if these demographics were not collected** | **N/A** |
| If collected and within the bounds of privacy constraints report on age, sex, gender and ethnicity for all study participants. | In Materials and Methods/Participants/ Table 1  “Sample characteristics by age group” |  |

**Design:**

| **Study protocol** | **Indicate where provided: section/figure legend** | **N/A** |
| --- | --- | --- |
| If the study protocol has been pre-registered, provide DOI. For clinical trials, provide the trial registration number OR cite DOI. |  | N/A |
|  |  |  |
| **Laboratory protocol** | **Indicate where provided: section/figure legend** | **N/A** |
| Provide DOI OR other citation details if detailed step-by-step protocols are available. |  | N/A |
|  |  |  |
| **Experimental study design (statistics details) *** | | |
| **For in vivo studies: State whether and how the following have been done** | **Indicate where provided: section/figure legend. If it could have been done, but was not, write “not done”** | **N/A** |
| Sample size determination |  | N/A |
| Randomisation |  | N/A |
| Blinding |  | N/A |
| Inclusion/exclusion criteria |  | N/A |
|  |  |  |
| **Sample definition and in-laboratory replication** | **Indicate where provided: section/figure legend** | **N/A** |
| State number of times the experiment was replicated in the laboratory. |  | N/A |
| Define whether data describe technical or biological replicates. |  | N/A |
|  |  |  |
| **Ethics** | **Indicate where provided: section/submission form** | **N/A** |
| Studies involving human participants: State details of authority granting ethics approval (IRB or equivalent committee(s), provide reference number for approval. | All participants or their legal guardians gave written informed consent prior to participation. The study was approved by the ethics committee of the Goethe University Frankfurt am Main (approval E 145/18). |  |
| Studies involving experimental animals: State details of authority granting ethics approval (IRB or equivalent committee(s), provide reference number for approval. |  | N/A |
| Studies involving specimen and field samples: State if relevant permits obtained, provide details of authority approving study; if none were required, explain why. |  | N/A |
|  |  |  |
| **Dual Use Research of Concern (DURC)** | **Indicate where provided: section/submission form** | **N/A** |
| If study is subject to dual use research of concern regulations, state the authority granting approval and reference number for the regulatory approval. |  | N/A |

**Analysis:**

| **Attrition** | **Indicate where provided: section/figure legend** | **N/A** |
| --- | --- | --- |
| Describe whether exclusion criteria were pre-established. Report if sample or data points were omitted from analysis. If yes, report if this was due to attrition or intentional exclusion and provide justification. | 1.Fourteen children were excluded due to : (i) incomplete task execution and missing data (n = 2); (ii) poor quality of the data (n = 7); (iii) technical issues during data acquisition (n = 5). Seven young adult participants were excluded due to incomplete task execution and missing data (n = 5) or being identified as extreme outlier (n = 2) based on interquartile range (IQR; above Q3_upper quartile(75th percentile)_ + 3xIQR or below Q1_lower quartile(25th percentile)_ – 3xIQR (Hawkins, 1980)) for memory behavioural measures. The excluded participants were comparable in terms of age, sex, and socio-economic status to the final sample.  2. An additional analysis on a subsample that included only participants who needed two learning cycles to reach the learning criterion was conducted. Twenty-one child participants were excluded, resulting in the final subsample of n = 28 children. The results from this subsample fully replicated the findings from the full sample, indicating that the amount of re-exposure to stimuli during encoding did not affect consolidation-related changes in memory retrieval at the behavioral level.  3. For fMRI analysis, single runs were excluded if there was (i) root-mean-square realignment estimates(Jenkinson et al., 2002) exceeding 1mm; and (ii) framewise displacement (FD) > 1, and (iii) less than two correct trials in the entire run. Based on these criteria, 14 single runs and two complete sessions in children were excluded from further analysis. |  |
|  |  |  |
| **Statistics** | **Indicate where provided: section/figure legend** | **N/A** |
| Describe statistical tests used and justify choice of tests. | 1.We conducted a linear mixed-effect model (LME model) using the lmer function from the lme4 package in R (Bates et al., 2015) and lmerTest (Kuznetsova et al., 2017). All LME models were calculated with maximum-likelihood estimation and Subject as the random intercept to account for between-subject variability. The LME models were applied for memory measures, univariate activation differences, representational similarity outputs. All p-values were FDR-adjusted for multiple comparisons due to multiple ROIs. Degrees of freedom were adjusted using the Satterthwaite’s method (Kuznetsova et al., 2017) if the assumptions of homogeneity of variances were violated. Significant effects were followed up with Sidak post-hoc multiple comparisons. For further group differences in socio-demographic measures, we performed one-way independent analysis of variance (ANOVA) or Games-Howell test (S. Lee & Lee, 2018). The effect size estimation was performed using omega squared (w^2^) as a less biased estimate for reporting practical significance of observed effects (Okada, 2013). To determine the amount of variance explained by the model, we used partR2 package (Stoffel et al., 2021).  2. Anatomical and functional MR data was pre-processed using fMRIPrep 22.0.0 (Esteban et al., 2019), based on Nipype 1.8.3 (Gorgolewski et al., 2011).  3. For each participant’s fMRI data, a first-level analysis was performed separately for each run using a general linear model (GLM) with eight experimental regressors. The regressors represented the onset and duration of the following events: (i) object recent_correct_, (ii) object remote_correct_, (iii) scene recent_correct_, (iv) scene remote_correct_, (v) object recent_incorrect_, (vi) object remote_incorrect_, (vii) scene recent_incorrect_, (viii) scene remote_incorrect_. The duration of object events was two seconds, while the duration of scene events was dependent on the reaction time (RT). The regressors were convolved with a hemodynamic response function, modelled with a double-gamma function with first and second derivatives. Confounding regressors were also included in the GLM and were calculated with fMRIPrep, namely six rigid body realignment parameters, framewise displacement, and standardised DVARS (D, temporal derivatives over time courses; VARS, variance over voxels). In addition, six anatomic component-based noise correction (CompCor, a combination of cerebrospinal fluid and white matter) regressors, global signal and cosine drift terms were included, based on previous methodological studies (Ciric et al., 2017; Esteban et al., 2020; Jones et al., 2021; Satterthwaite et al., 2013). The functional images were spatially smoothed with SUSAN (Smallest Univalue Segment Assimilating Nucleus, Smith & Brady, (1997)), applying a Gaussian kernel with a full-width at half-maximum of 6 mm. A high-pass Gaussian filter with a cut-off period of 80 s was applied. Contrasts were defined for each run per subject, and within-subject fixed-effects averaging across runs within each session was conducted per subject. Group-level analysis was performed with FLAME1 (Woolrich et al., 2004) within each session, based on the statistical maps obtained from the first-level analysis. The main contrast of interest was *object remote > object recent*, as we were primarily interested in the reinstatement of object-scene association before the scene was shown. Univariate analysis was performed with statistical tests voxel-wise and corrected for multiple comparisons with cluster-based thresholding using a z threshold of z > 3.1 and a two-tailed probability of .001.  4. For the multivariate analysis, single-event (i.e., for every event on each trial) β (beta) estimates were first computed by modelling BOLD time course with a series of Generalized Linear Models (GLM) using the Least Square Separate method (LSS; Abdulrahman & Henson, 2016; Mumford et al., 2012). Each trial contained three events (i.e., object, fixation, and scene), hence a total of 30 GLMs (i.e., ten for objects, ten for fixations, and ten for scenes) were computed for each run, session, and participant. Each of the GLMs contained four experimental regressors: for instance, one for the single fixation of interest and three more for the rest of the events (i.e., for all other fixations except the fixation of interest, for all objects, and for all scenes). The same set up was followed for the object GLMs and the scene GLMs. The regressors were convolved with the hemodynamic response function, which was modelled with a double-gamma function with first and second derivatives. Additionally, the same confounding regressors as the ones for mean-activation analysis were included.  Beta estimates were obtained from a Least Square Separate (LSS) regression model. Each event was modeled with their respective onset and duration and, as such, one beta value was estimated per event (with the lags between events differing from trial to trial). The jitter was included to enable an estimation of the patterns evoked by the events and all subsequent RSA analyses were conducted normally on these estimates without further controls.  5. To examine the connections between brain function and behavior, we utilized brain metrics generated via the application of a multivariate method known as Partial Least Square Correlation (PLSC) (Abdi & Williams, 2013; McIntosh et al., 1996; Schommartz et al., 2023). This approach focuses on multivariate links between specified neural measures in Regions of Interest (ROIs) and fluctuations in memory performance over short and long delays across different age cohorts. We argue that this multivariate strategy offers a more comprehensive understanding of the relationships between brain metrics across various ROIs and memory performance, given their mutual dependence and connectivity (refer to Genon et al. (2022) for similar discussions). |  |
|  |  |  |
| **Data availability** | **Indicate where provided: section/submission form** | **N/A** |
| For newly created and reused datasets, the manuscript includes a data availability statement that provides details for access (or notes restrictions on access). | In Data availability statement/ All behavioural and neuroimaging data has been deposited at https://osf.io/grku2/ and are publicly available. |  |
| When newly created datasets are publicly available, provide accession number in repository OR DOI and licensing details where available. | Schommartz, I. (2025, October 30). Recent and remote memory consolidation. Date Set. Retrieved from osf.io/grku2  Doi: [**10.17605/OSF.IO/GRKU2**](https://doi.org/10.17605/OSF.IO/GRKU2) |  |
| If reused data is publicly available provide accession number in repository OR DOI, OR URL, OR citation. |  | N/A |
|  |  |  |
| **Code availability** | **Indicate where provided: section/figure legend** | **N/A** |
| For any computer code/software/mathematical algorithms essential for replicating the main findings of the study, whether newly generated or re-used, the manuscript includes a data availability statement that provides details for access or notes restrictions. | In Data Availably Statement/ The code for the analyses presented in this paper is openly accessible at <https://github.com/iryna1schommartz/memokid_fmri>. |  |
| Where newly generated code is publicly available, provide accession number in repository, OR DOI OR URL and licensing details where available. State any restrictions on code availability or accessibility. | <https://github.com/iryna1schommartz/memokid_fmri>. |  |
| If reused code is publicly available provide accession number in repository OR DOI OR URL, OR citation. |  | N/A |

**Reporting:**

The MDAR framework recommends adoption of discipline-specific guidelines, established and endorsed through community initiatives.

| **Adherence to community standards** | **Indicate where provided: section/figure legend** | **N/A** |
| --- | --- | --- |
| State if relevant guidelines (e.g., ICMJE, MIBBI, ARRIVE, STRANGE) have been followed, and whether a checklist (e.g., CONSORT, PRISMA, ARRIVE) is provided with the manuscript. |  | N/A |

* We provide the following guidance regarding transparent reporting and statistics; we also refer authors to [Ten common statistical mistakes to watch out for when writing or reviewing a manuscript](https://doi.org/10.7554/eLife.48175).

**Sample-size estimation**

- You should state whether an appropriate sample size was computed when the study was being designed
- You should state the statistical method of sample size computation and any required assumptions
- If no explicit power analysis was used, you should describe how you decided what sample (replicate) size (number) to use

**Replicates**

- You should report how often each experiment was performed
- You should include a definition of biological versus technical replication
- The data obtained should be provided and sufficient information should be provided to indicate the number of independent biological and/or technical replicates
- If you encountered any outliers, you should describe how these were handled
- Criteria for exclusion/inclusion of data should be clearly stated
- High-throughput sequence data should be uploaded before submission, with a private link for reviewers provided (these are available from both GEO and ArrayExpress)

**Statistical reporting**

- Statistical analysis methods should be described and justified
- Raw data should be presented in figures whenever informative to do so (typically when N per group is less than 10)
- For each experiment, you should identify the statistical tests used, exact values of N, definitions of center, methods of multiple test correction, and dispersion and precision measures (e.g., mean, median, SD, SEM, confidence intervals; and, for the major substantive results, a measure of effect size (e.g., Pearson's r, Cohen's d)
- Report exact p-values wherever possible alongside the summary statistics and 95% confidence intervals. These should be reported for all key questions and not only when the p-value is less than 0.05.

**Group allocation**

- Indicate how samples were allocated into experimental groups (in the case of clinical studies, please specify allocation to treatment method); if randomization was used, please also state if restricted randomization was applied
- Indicate if masking was used during group allocation, data collection and/or data analysis
