# Supplementary material for: Neural correlates and reinstatement of recent and remote memory in children and young adults
Source: eLife. 2025 Dec 5;12:RP89908. doi: 10.7554/eLife.89908 (PMC12680376; doi:10.7554/eLife.89908)
Supplement: Supplementary file 1. [file elife-89908-supp1.docx]

Supplementary File 1

*Statistical overview of the linear mixed effects model for memory retention rates for initially correctly learned items (corrected for chance performance) based on participants who needed only two learning cycles (N = 28).*

|  | 1. **Recent Memory Retention** | | | **(B) Overall Memory Retention** | |
| --- | --- | --- | --- | --- | --- |
| *Predictors* | *F-value_(DenDF)_* | | *p-value* | *F-value_(DenDF)_* | *p-value* |
|  | 4.64_(1,55)_  30.16_(1,59)_  .02_(1,60)_  4.01_(1,60)_  1.18_(1,55)_ | |  |  |  |
| Session |  |  | **.035** |  |  |
| Group |  |  | **<.001** | 33.53_(1,65)_ | **<.001** |
| Item Type |  |  |  | **230.02** _(3,192)_ | **<.001** |
| IQ |  |  | .885 | 1.49_(1,66)_ | **.226** |
| Sex |  |  | .049 | 4.42_(1,66)_ | .**039** |
| Session x Group |  |  | .281 |  |  |
| Item Type x Group |  | |  | 10.56_(3,192)_ | **<.001** |
| **Random Effects** |  |  |  |  |  |
| σ^2^ | 32.92 |  |  | 42.11 |  |
| τ_00_ _subNo_ | 29.74 |  |  | 16.45 |  |
| ICC | .47 |  |  | .28 |  |
| N _subNo_ | 67 |  |  | 67 |  |
| Observations | 122 |  |  | 258 |  |
| Marginal R^2^ / Conditional R^2^ | 0.331/ 0.649 |  |  | .697/.782 |  |

*Notes.* Subject was included as random intercept. Group (children and young adults), Session (Day 1, Day 14 and Day1 and Day14 _after 30 minutes_), Item Type (baseline_learning_, immediate, recent vs remote) were included as fixed effects. IQ, Sex, Handedness were included as covariates. ^a^The following reference levels where used: for Session, Day 1/14; for Group, Children; for Item Type, baseline; for Sex, male; for Handedness, right-side handedness. IQ = Intelligence Quotient; σ2 – residuals, τ00 – variance of the random intercept. Type III Analysis of Variance Table with Satterthwaite's method.
