# Supplementary material for: Neural correlates and reinstatement of recent and remote memory in children and young adults
Source: eLife. 2025 Dec 5;12:RP89908. doi: 10.7554/eLife.89908 (PMC12680376; doi:10.7554/eLife.89908)
Supplement: Supplementary file 2. [file elife-89908-supp2.docx]

Supplementary File 2

*Statistical overview of post hoc analysis of the Item Type x Group Interaction effects for the linear mixed effects model for memory retention rates for initially correctly learned items (corrected for chance performance) based on participants who needed only two learning cycles.*

| Contrast | Estimate | df | t | p-value |
| --- | --- | --- | --- | --- |
| d0 vs d1/14 recent CH vs. YA | 10.86 | 197 | 4.701 | <.001 |
| d0 vs d1 remote CH vs. YA | 8.67 | 201 | 3.720 | .003 |
| d0 vs d14 remote CH vs. YA | 11.41 | 201 | 4.736 | <.001 |
| d1/14 vs d1/14 CH | 14.857 | 216.182 | 8.820 | .000 |
| d1/14 vs d1/14 YA | 1.913 | 216.182 | 1.199 | .968 |
| d0 vs d1/d14 recent CH | 12.77 | 197 | 7.248 | <.001 |
| d0 vs d1/d14 recent YA | 1.91 | 197 | 1.28 | .946 |
| d0 vs d1 remote CH | 14.00 | 198 | 7.859 | <.001 |
| d0 vs d1 remote YA | 5.33 | 198 | 3.53 | .006 |
| d0 vs d14 remote CH | 35.75 | 201 | 19.34 | <.001 |
| d0 vs d14 remote YA | 24.4 | 200 | 15.77 | <.001 |

*Notes.* d – Day; CH – children; YA – young adults; df – degrees of freedom, t – t-test; All post hoc test were Sidak corrected.
