# Supplementary material for: Neural correlates and reinstatement of recent and remote memory in children and young adults
Source: eLife. 2025 Dec 5;12:RP89908. doi: 10.7554/eLife.89908 (PMC12680376; doi:10.7554/eLife.89908)
Supplement: Supplementary file 3. [file elife-89908-supp3.docx]

Supplementary File 3

Two-sided permutation t-tests were conducted to assess whether the mean signal difference of the contrast **remote > recent** significantly differed from zero for each combination of ROI, session, and group. For each subset, the mean signal difference value, t-statistic, and unadjusted and FDR-adjusted p-values are reported. P-values were corrected for multiple comparisons using the False Discovery Rate (FDR) method.

| **ROI** | **Session** | **Group** | **Mean value** | **t statistic** | **p-value** | **Adjusted**  **p-value** |
| --- | --- | --- | --- | --- | --- | --- |
| **Children – Short Delay** | | | | | | |
| Medial Prefrontal Cortex | Day 1 | Children | .056 | 2.007 | .051 | .102 |
| Cerebellum | Day 1 | Children | **.047** | **2.937** | **.005** | **.017** |
| Retrosplenial Cortex | Day 1 | Children | .046 | 2.437 | .019 | .054 |
| Precuneus | Day 1 | Children | .019 | .920 | .362 | .451 |
| Hippocampus Anterior | Day 1 | Children | .027 | 2.034 | .048 | .102 |
| Hippocampus Posterior | Day 1 | Children | .018 | 1.301 | .200 | .307 |
| Parahippocampus Anterior | Day 1 | Children | .007 | .456 | .651 | .723 |
| Parahippocampus Posterior | Day 1 | Children | **.072** | **3.719** | **<.001** | **.002** |
| Ventrolateral Prefrontal Cortex | Day 1 | Children | .018 | .881 | .383 | .451 |
| Lateral Occipital Cortex | Day 1 | Children | .018 | 1.009 | .318 | .433 |

| **Young Adults – Short Delay** | | | | | | |
| --- | --- | --- | --- | --- | --- | --- |
| Medial Prefrontal Cortex | Day 1 | Young Adults | .003 | .121 | .902 | .927 |
| Cerebellum | Day 1 | Young Adults | .028 | 2.396 | .022 | .058 |
| Retrosplenial Cortex | Day 1 | Young Adults | -.010 | -.882 | .383 | .451 |
| Precuneus | Day 1 | Young Adults | **-.062** | **-4.196** | **<.001** | **.001** |
| Hippocampus Anterior | Day 1 | Young Adults | .028 | 2.169 | .036 | .086 |
| Hippocampus Posterior | Day 1 | Young Adults | .012 | 1.526 | .068 | .135 |
| Parahippocampus Anterior | Day 1 | Young Adults | .019 | 1.539 | .326 | .433 |
| Parahippocampus Posterior | Day 1 | Young Adults | **.063** | **5.431** | **<.001** | **<.001** |
| Ventrolateral Prefrontal Cortex | Day 1 | Young Adults | **.129** | **6.455** | **<.001** | **<.001** |
| Lateral Occipital Cortex | Day 1 | Young Adults | .022 | 1.387 | .173 | .277 |

| **Children – Long Delay** | | | | | | |
| --- | --- | --- | --- | --- | --- | --- |
| Medial Prefrontal Cortex | Day 14 | Children | **.103** | **3.252** | **.002** | **.009** |
| Cerebellum | Day 14 | Children | .046 | 1.586 | .120 | .229 |
| Retrosplenial Cortex | Day 14 | Children | -.031 | -1.393 | .171 | .277 |
| Precuneus | Day 14 | Children | **-.063** | **-2.609** | **.013** | **.039** |
| Hippocampus Anterior | Day 14 | Children | .009 | .376 | .709 | .758 |
| Hippocampus Posterior | Day 14 | Children | .007 | .361 | .720 | .758 |
| Parahippocampus Anterior | Day 14 | Children | .027 | .995 | .282 | .402 |
| Parahippocampus Posterior | Day 14 | Children | .047 | 2.023 | .050 | .102 |
| Ventrolateral Prefrontal Cortex | Day 14 | Children | **.073** | **3.055** | **.004** | **.014** |
| Lateral Occipital Cortex | Day 14 | Children | .049 | 2.281 | .028 | .069 |

| **Young Adults – Long Delay** | | | | | | |
| --- | --- | --- | --- | --- | --- | --- |
| Medial Prefrontal Cortex | Day 14 | Young Adults | .002 | .071 | .944 | .944 |
| Cerebellum | Day 14 | Young Adults | **.113** | **6.216** | **<.001** | **<.001** |
| Retrosplenial Cortex | Day 14 | Young Adults | .021 | 1.270 | .213 | .315 |
| Precuneus | Day 14 | Young Adults | **-.089** | **-4.046** | **<.001** | **.001** |
| Hippocampus Anterior | Day 14 | Young Adults | .014 | .977 | .336 | .433 |
| Hippocampus Posterior | Day 14 | Young Adults | .005 | .580 | .566 | .646 |
| Parahippocampus Anterior | Day 14 | Young Adults | .015 | 1.095 | .141 | .235 |
| Parahippocampus Posterior | Day 14 | Young Adults | **.147** | **8.058** | **<.001** | **<.001** |
| Ventrolateral Prefrontal Cortex | Day 14 | Young Adults | **.242** | **9.325** | **<.001** | **<.001** |
| Lateral Occipital Cortex | Day 14 | Young Adults | **.148** | **6.287** | **<.001** | **<.001** |

*Notes*. ROI – region of interest; p – p-value; *p < .05; ** < .01, *** < .001 (significant difference).
