# Supplementary material for: Neural correlates and reinstatement of recent and remote memory in children and young adults
Source: eLife. 2025 Dec 5;12:RP89908. doi: 10.7554/eLife.89908 (PMC12680376; doi:10.7554/eLife.89908)
Supplement: Supplementary file 4. [file elife-89908-supp4.docx]

Supplementary File 4

*Test of neural activation during object presentation separately for recent and remote memories for significance (higher than zero).*

|  | Recent | | | | | Remote | | |
| --- | --- | --- | --- | --- | --- | --- | --- | --- |
|  | Young Adults | | | | | | | |
| ROI | *Day* | *mean* | *T test* | *p_(FDRadj)_* | *mean* | | *T test* | *p_(FDRadj)_* |
| Hippocampus Anterior | Day 1 | .054 | 3.76 | **<.001** | .083 | 6.42 | | **<.001** |
|  | Day 14 | .072 | 6.25 | **<.001** | .089 | 6.95 | | **<.001** |
| Hippocampus Posterior | Day 1 | .056 | 5.79 | **<.001** | .069 | 6.91 | | **<.001** |
|  | Day 14 | .063 | 7.71 | **<.001** | .068 | 6.66 | | **<.001** |
| Parahippocampal Gyrus Anterior | Day 1 | .025 | 1.98 | **.031** | .044 | 3.45 | | **.001** |
|  | Day 14 | .038 | 2.59 | **.010** | .054 | 4.31 | | **<.001** |
| Retrosplenial Cortex | Day1 | .120 | 6.75 | **<.001** | .113 | 5.64 | | **<.001** |
|  | Day14 | .079 | 5.39 | **<.001** | .108 | 7.24 | | **<.001** |
| Precuneus | Day1 | .099 | 5.37 | **<.001** | .034 | 1.808 | | **.041** |
|  | Day14 | .150 | 8.48 | **<.001** | .057 | 3.79 | | **<.001** |
|  | Children | | | | | | | |
|  |  | *mean* | *T test* | *p_(FDRadj)_* | *mean* | *T Test* | | *p_(FDRadj)_* |
| Hippocampus Anterior | Day 1 | .043 | 2.51 | **.011** | .076 | 4.47 | | **<.001** |
|  | Day 14 | .080 | 4.09 | **<.001** | .092 | 3.37 | | **.001** |
| Hippocampus Posterior | Day 1 | .017 | 1.09 | .141 | **.037** | **2.405** | | **.013** |
|  | Day 14 | .035 | 2.28 | **.016** | .048 | 2.45 | | **.013** |
| Parahippocampal Gyrus Anterior | Day 1 | .058 | 3.69 | **.001** | .064 | 3.81 | | **.000** |
|  | Day 14 | .070 | 2.64 | **.009** | .098 | 3.14 | | **.002** |
| Retrosplenial Cortex | Day1 | .042 | 1.97 | **.031** | .089 | 4.28 | | **<.001** |
|  | Day14 | .090 | 4.59 | **<.001** | .055 | 2.37 | | **.014** |
| Precuneus | Day1 | .036 | 1.63 | .056 | .052 | 1.31 | | **.013** |
|  | Day14 | .130 | 6.72 | **<.001** | .053 | 1.88 | | **.037** |

*Notes*.To test for significance we used one-sample permutation t-test for more robust calculations with Monte-Carlo permutation percentile confidence interval. All p-values for False Discovery Rate (FDR) corrected for multiple comparisons. ROI – region of interest; p – p-value; FDRadj – False Discovery Rate adjustment; *p < .05; ** < .01, *** < .001 (significant difference).
