# Supplementary material for: Neural correlates and reinstatement of recent and remote memory in children and young adults
Source: eLife. 2025 Dec 5;12:RP89908. doi: 10.7554/eLife.89908 (PMC12680376; doi:10.7554/eLife.89908)
Supplement: Supplementary file 5. [file elife-89908-supp5.docx]

Supplementary File 5

*Full statistical overview of LME model for univariate analysis (includes only ROI that show above or below zero upregulation).*

|  | **Main Effect**  **of Group** | | **Main Effect**  **of Session** | | **Group x Session Interaction** | |  |
| --- | --- | --- | --- | --- | --- | --- | --- |
| ***Regions of Interest*** | *F_(DF)_* | *p* | *F_(DF)_* | *p* | *F_(DF)_* | p | *R2* |
| Parahippocampal Gyrus Posterior | 2.97_(1,83)_ | .088_(.106)_ | 2.48_(1,100)_ | .118_(.142)_ | 9.54_(1,83)_ | **.002_(.012)_** | .200⊥ |
| Medial Prefrontal Cortex | 7.61_(1,86)_ | **.007_(.014)_** | .42_(1,99)_ | .517_(.517)_ | 1.16_(1,83)_ | .284_(.284)_ | .369⊥ |
| Ventrolateral Prefrontal Cortex | 31.35_(1,82)_ | **<.001_(<.001)_** | 10.68_(1,99)_ | **.001_(.003)_** | 1.61_(1,83)_ | .207_(.248)_ | .309⊥ |
| Cerebellum | 1.54_(1,161)_ | .215_(.215)_ | 4.67_(1,161)_ | .036_(.054_**_)_** | 7.68_(1,161)_ | **.006_(.018)_** | .100∩ |
| Precuneus | 5.09_(1,161)_ | **.025_(.037)_** | 6.50_(1,161)_ | **.011_(.022)_** | 1.61_(1,161)_ | .205_(.248)_ | .099∩ |
| Lateral Occipital Cortex | 9.12_(1,82)_ | **.003_(.009)_** | 16.76_(1,97)_ | **<.001_(<.001)_** | 6.42_(1,81)_ | **.013_(.026)_** | .324⊥ |

|  | **Main Effect**  **of Sex** | | **Main Effect**  **of Handedness** | | **Main Effect**  **of IQ** | | **Main Effect**  **Of Reaction Time** | |
| --- | --- | --- | --- | --- | --- | --- | --- | --- |
| ***Regions of Interest*** | *F_(DF)_* | *p* | *F_(DF)_* | *p* | *F_(DF)_* | p | *F_(DF)_* | *p* |
| Parahippocampal Gyrus Posterior | 1.26_(1,84)_ | .263 | .03_(1,93)_ | .962 | 1.28_1,84)_ | **.**259 | .09_(1,155)_ | .764 |
| Medial Prefrontal Cortex | .62_(1,87)_ | .430 | .50_(1,95)_ | .607 | 5.16_(1,87)_ | **.024** | .22_(1,160)_ | .635 |
| Ventrolateral Prefrontal Cortex | 1.11_(1,83)_ | .294 | .71_(1,92)_ | .494 | .09_(1,83)_ | .764 | .20_(1,154)_ | .654 |
| Cerebellum | 3.15_(1,161)_ | .077 | .21_(1,161)_ | .806 | .781_(1,161)_ | .378 | .11_(1,161)_ | .741 |
| Precuneus | .35_(1,161)_ | .553 | .20_(1,161)_ | .817 | .08_(1,161)_ | .776 | .137_(1,161)_ | .712 |
| Lateral Occipital Cortex | .10_(1,83)_ | .752 | .76_(1,92)_ | .468 | 3.04_(1,83)_ | .084 | .005_(1,159)_ | .944 |

*Notes.* *Notes.* Subject was included as random effect. Group (children, young adults), Session (Day 1 remote > recent, Day 14 remote > recent), and their interaction were included as fixed effect. The following reference levels where used: for Session – Day 1; for Group – Children; F – F-value; DF – degrees of freedom; p – p-value; FDR_adj – False Discovery Rate adjusted; R2 – amount of variance explained by the model (∩- marginal; ⊥ - conditional). Type III Analysis of Variance Table with Satterthwaite’s method. *p < .05; ** < .01, *** < .001 (significant difference). All p-values of main and interactions effects were FDR-adjusted for multiple comparisons.
