# Supplementary material for: Neural correlates and reinstatement of recent and remote memory in children and young adults
Source: eLife. 2025 Dec 5;12:RP89908. doi: 10.7554/eLife.89908 (PMC12680376; doi:10.7554/eLife.89908)
Supplement: Supplementary file 6. [file elife-89908-supp6.docx]

**Supplementary File 6**

*Statistical overview of the main and interaction effects of the linear mixed effects models recent and remote univariate results of correctly recognized items.*

|  | **Hippocampus Anterior** | | **Hippocampus Posterior** | | **Parahippocampal Cortex Anterior** | |
| --- | --- | --- | --- | --- | --- | --- |
|  | *F_(DF)_* | *p-value* | *F_(DF)_* | *p-value* | *F_(DF)_* | *p-value* |
| Delay: (Recent > Remote) | 3.62_(231)_ | .058 | 2.17_(234)_ | .142 | 1.70_(227)_ | .194 |
| Group: (Adults > Children) | .01_(80)_ | .912(.912) | **5.62_(85)_** | **.020(.060)** | 4.77_(76)_ | .030(.060) |
| Session: (Day1> Day14) | 2.65_(253)_ | .106 | 1.24_(253)_ | .267 | 1.48_(250)_ | .225 |
| Delay x Group | .001_(253)_ | .977 | .155_(234)_ | .694 | .000_(227)_ | .994 |
| Delay x Session | .462_(253)_ | .490 | .172_(234)_ | .679 | .110_(227)_ | .740 |
| Group x Session | .486_(253)_ | .486 | .670_(253)_ | .414 | .206_(250)_ | .650 |
| Delay x Group x Session | .022_(231)_ | .880 | .000_(234)_ | .990 | .215_(222)_ | .643 |
|  | **Medial Prefrontal Cortex** | | **Precuneus** | | **Retrosplenial Cortex** | |
| Delay: (Recent > Remote) | 6.82_(235)_ | .009_()_ | 15.14_(235)_ | .0001_()_ | .49_(229)_ | .484 |
| Group: (Adults > Children) | 7.86_(85)_ | .006_()_ | .959_(84)_ | **.303_(.)_** | 3.22_(80)_ | **.076(.101)** |
| Session: (Day1> Day14) | 17.74_(256)_ | <.001 | 9.49_(256)_ | .002 | .306_(247)_ | .581 |
| Delay x Group | 6.89 _(235)_ | .009 | 2.87_(235)_ | .091 | .034_(229)_ | .853 |
| Delay x Session | .873_(235)_ | .351_()_ | 4.74_(235)_ | .030_(.)_ | .879_(229)_ | .340 |
| Group x Session | 6.75_(256)_ | .009 | .196_(256)_ | .657 | 2.07_(248)_ | .152 |
| Delay x Group x Session | .477_(235)_ | .490_()_ | 1.29_(233)_ | .256_(.)_ | 5.756_(229)_ | .017 |
|  | **Ventrolateral Prefrontal Cortex** | | **Cerebellum** | | **Parahippocampal Cortex Posterior** | |
| Delay: (Recent > Remote) | 80.52_(231)_ | <.001_()_ | 28.66_(232)_ | <.001_(<.001)_ | 50.25_(233)_ | <.001_(<.001)_ |
| Group: (Adults > Children) | 5.43_(83)_ | .022_()_ | .808_(81)_ | .371_()_ | 1.55_(84)_ | .217_()_ |
| Session: (Day1> Day14) | 11.52_(248)_ | <.001 | .002_(254)_ | .965 | 34.79_(251)_ | <.001 |
| Delay x Group | 27.45_(231)_ | <.001 | .783_(232)_ | .377 | 2.59_(233)_ | .109 |
| Delay x Session | 10.82_(231)_ | .001_()_ | 4.29_(232)_ | .039_()_ | 1.87_(233)_ | .172_()_ |
| Group x Session | .004_(248)_ | .948 | 1.24_(248)_ | .266 | .671_(251)_ | .414 |
| Delay x Group x Session | 1.275_(231)_ | .260_()_ | 3.99_(232)_ | .047_()_ | 4.218_(233)_ | .041_()_ |
|  | **Lateral Occipital Cortex** | |  | | | |
| Delay: (Recent > Remote) | 21.44_(233)_ | <.001_(<.001)_ |  |  |  |  |
| Group: (Adults > Children) | 30.07_(86)_ | <.001 |  |  |  |  |
| Session: (Day1> Day14) | 46.66_(246)_ | <.001 |  |  |  |  |
| Delay x Group | 3.28_(233)_ | .071 |  |  |  |  |
| Delay x Session | 7.46_(233)_ | .007_()_ |  |  |  |  |
| Group x Session | 1.18_(246)_ | .278 |  |  |  |  |
| Delay x Group x Session | 1.29_(233)_ | .257_(.)_ |  |  |  |  |

*Notes.* Subject was included as a random effect. Group (children, young adults), Delay ( recent, remote), Session (Day1, Day 14), and their interaction were included as fixed effect. The following reference levels where used: for Delay, recent; for Group, Children; F – F-value; DF – degrees of freedom; p – p-value; Type III Analysis of Variance Table with Satterthwaite's method. *p < .05; ** <.01, ***<.001 (significant difference).
