# Supplementary material for: Neural correlates and reinstatement of recent and remote memory in children and young adults
Source: eLife. 2025 Dec 5;12:RP89908. doi: 10.7554/eLife.89908 (PMC12680376; doi:10.7554/eLife.89908)
Supplement: Supplementary file 7. [file elife-89908-supp7.docx]

**Supplementary File 7**

**fMRI univariate analysis**

*Regions exhibiting stronger activation for remote vs. recent items in (i) young adults, (ii) children, (iii) children vs young adults, and (iv) young adults vs children on Day 1 (short delay). To capture the involved brain region better, local maxima are presented in addition to cluster maxima for the largest clusters. The preprocessing steps included global signal regression.*

| **Day 1 (Short Delay)** | | | | |  |
| --- | --- | --- | --- | --- | --- |
| **Young adults** | | | | | |
| **Region** | **x** | **y** | **x** | **Z-max** | **# voxels** |
| Left Middle Frontal Gyrus | - 44 | 2 | 40 | 6.67 | 2990 |
| Left Insula Cortex | - 34 | 22 | 2 | 6.58 |  |
| Left Inferior Frontal Gyrus, Pars Opercularis | - 44 | 6 | 34 | 6.03 |  |
| Left Lateral Occipital Cortex | - 28 | - 76 | 36 | 6.82 | 2272 |
| Left Superior Parietal Lobule | - 34 | - 50 | 44 | 5.11 |  |
| Left Fusiform Gyrus | - 44 | - 60 | - 12 | 6.7 | 1661 |
| Left Parahippocampal Gyrus | - 34 | - 34 | - 16 | 4.58 |  |
| Right Cerebellum | 30 | - 60 | - 28 | 6.03 | 1049 |
| Right Lateral Occipital Cortex | 34 | - 72 | 40 | 5.96 | 943 |
| Right Inferior Parietal Lobule | 38 | - 78 | 26 | 4.3 |  |
| Right Parahippocampal Gyrus | 32 | - 34 | - 16 | 5.29 | 718 |
| Right Inferior Temporal Gyrus | 52 | - 54 | - 10 | 5.17 |  |
| Left Superior Frontal Gyrus | - 4 | 16 | 48 | 5.04 | 405 |
| Right insular cortex | 30 | 24 | 2 | 5.25 | 279 |
| Right Middle Frontal Gyrus, Pars Triangularis | 40 | 30 | 20 | 3.61 |  |
| Right precentral Gyrus | 42 | 2 | 30 | 4.97 | 146 |
| Right Middle Frontal Gyrus, Pars Opercularis | 50 | 16 | 32 | 3.41 |  |
| Left Frontal Orbital Cortex | - 26 | 32 | - 10 | 4.51 | 123 |
| Left Cingulate Gyrus | - 4 | 2 | 28 | 4.86 | 103 |
|  | | | | | |
| **Children** | | | | | |
| Right Temporal Occipital Fusiform Cortex | 26 | - 44 | - 8 | 5.1 | 658 |
| Right Parahippocampal Gyrus | 30 | - 36 | - 16 | 4.93 |  |
| Right Precuneus | 8 | - 52 | 6 | 4.79 |  |
| Left Temporal Fusiform Gyrus | - 34 | - 42 | - 12 | 5.59 | 500 |
| Left Parahippocampal Gyrus | - 18 | - 42 | - 10 | 4.91 |  |
| Left Precuneus Cortex | - 14 | - 60 | 10 | 4.47 | 160 |
| Left Lateral Occipital Cortex | - 36 | - 84 | 26 | 4.95 | 112 |
|  |  |  |  |  |  |
| **Children > Young Adults** | | | | | |
|  |  |  |  |  |  |
| Right precuneus | 4 | - 48 | 30 | 5.25 | 1051 |
| Left precuneus | - 4 | - 48 | 40 | 4.68 |  |
| Right Superior Parietal Lobule | 12 | - 32 | 50 | 4.99 | 203 |
| Right Parietal Operculum Cortex | 54 | - 30 | 24 | 3.32 | 149 |
|  |  |  |  |  |  |
| **Young Adults > Children** | | | | | |
|  |  |  |  |  |  |
| Left Precentral Gyrus, Middle Frontal Gyrus | - 44 | 2 | 40 | 4.8 | 501 |
| Left Inferior Frontal Gyrus | - 54 | 14 | 10 | 3.39 |  |
| Left Frontal Operculum Cortex | - 34 | 22 | 2 | 5.48 | 260 |
| Right Cerebellum | 12 | - 76 | - 20 | 4.7 | 141 |
| Left Medial Frontal Gyrus | - 2 | 16 | 48 | 4.2 | 118 |
| Left/Right Insular Cortex | 32 | 22 | 2 | 4.66 | 113 |
| Left/Right Lateral Occipital Cortex | - 26 | - 74 | 36 | 4.5 | 107 |

*Regions exhibiting stronger activation for remote vs. recent items in (i) young adults, (ii) children, (iii) children vs young adults, and (iv) young adults vs children on Day 14 (long delay). To capture the involved brain region better, local maxima are presented in addition to cluster maxima for the largest clusters. The preprocessing steps included global signal regression.*

| **Day 14 (Long Delay)** | | | | | |
| --- | --- | --- | --- | --- | --- |
| **Young Adults** | | | | | |
| **Region** | **x** | **y** | **x** | **Z-max** | **# voxels** |
| Left/Right Occipital Fusiform Gyrus | - 46 | - 58 | - 16 | 7.62 | 19227 |
| Left Lateral Occipital Cortex | - 30 | - 60 | - 14 | 7.25 |  |
| Left Middle Frontal Gyrus, Pars Opercularis, |  |  |  | 7.17 | 2890 |
| Left Superior Frontal Gyrus | - 6 | 12 | 56 | 6.78 |  |
| Right Inferior Frontal Gyrus, Pars Opercularis, Pars Trinagularis | 46 | 12 | 28 | 6 | 691 |
| Left Insular Cortex | - 32 | 22 | 2 | 6.7 | 501 |
| Left Caudate | - 10 | 4 | 10 | 5.58 | 456 |
| Right Frontal Orbital Cortex | 34 | 28 | 0 | 6.11 | 298 |
| Right Cerebellum | 16 | - 44 | - 46 | 4.97 | 250 |
| Right Caudate | 8 | 12 | 2 | 5.27 | 215 |
| Left Cerebellum | - 34 | - 68 | - 54 | 6.1 | 211 |
|  | | | | | |
| **Children** | | | | | |
| Left Temporal Fusiform Gyrus | - 34 | - 26 | - 24 | 4.91 | 580 |
| Left anterior Parahippocampal Gyrus, Hippocampus | - 36 | - 18 | - 24 | 4.4 |  |
| Left Lateral Occipital Cortex | - 48 | - 58 | - 16 | 4.25 |  |
| Right Temporal Occipital Fusiform Cortex | 40 | - 54 | - 18 | 4.34 | 448 |
| Right Lateral Occipital Cortex | 50 | - 70 | - 12 | 4.2 |  |
|  |  |  |  |  |  |
| **Children > Young Adults** | | | | | |
|  |  |  |  |  |  |
| Right/Left angular gyrus | 62 | - 40 | 44 | 4.8 | 847 |
| Right/Left Lateral Occipital Cortex | 46 | - 66 | 48 | 4.44 |  |
| Right Superior Frontal Gyrus | 20 | 30 | 58 | 4.58 | 640 |
| Right/Left Superior Temporal Gyrus |  |  |  | 4.73 | 493 |
| Right Precuneus | 8 | - 52 | 30 | 4.51 | 332 |
| Right Medial Frontal Cortex | 8 | 50 | - 2 | 4.35 | 287 |
| Right Middle Temporal Gyrus | 66 | - 18 | - 20 | 4.17 | 203 |
| Left Middle Frontal Gyrus | - 20 | 36 | 38 | 4.31 | 154 |
| Left Cingulate Gyrus | - 14 | - 50 | 30 | 4.36 | 138 |
|  |  |  |  |  |  |
| **Young Adults > Children** | | | | | |
|  |  |  |  |  |  |
| Right/Left Cerebellum | 14 | - 72 | - 22 | 5.77 | 3162 |
| Left Occipital Fusiform Gyrus | - 20 | - 90 | - 14 | 5.22 | 1229 |
| Left Lateral Occipital Cortex | - 30 | - 80 | 36 | 5.62 | 620 |
| Left Middle Frontal Gyrus, Inferior Frontal Gyrus | - 44 | 12 | 30 | 4.8 | 387 |
| Right Precuneus | 18 | - 58 | 20 | 4.39 | 205 |
| Left Superior Frontal Gyrus | - 6 | 12 | 56 | 5.12 | 165 |
| Left Posterior Parahippocampal Gyrus, Hippocampus | - 28 | - 32 | - 18 | 3.9 | 96 |

*Regions exhibiting stronger activation for remote vs. recent items that decreases over time (i) in young adults stronger than in children (ii) children stronger than in adults; that increases over time (iii) in young adults stronger than in children, and (iv) in children stronger than in young adults. To capture the involved brain region better, local maxima are presented in addition to cluster maxima for the largest clusters. The preprocessing steps included global signal regression.*

| **Decrease Across Time** | | | | | |
| --- | --- | --- | --- | --- | --- |
|  | | | | | |
| **Young Adults > Children** | | | | | |
|  | | | | | |
| **Region** | **x** | **y** | **x** | **Z-max** | **# voxels** |
| Right Superior Parietal Lobule, Agular Gyrus | 42 | - 50 | 58 | 3.69 | 946 |
| Right Middle Frontal Gyrus | 42 | 56 | 2 | 4.16 | 546 |
| Left Middle Frontal Gyrus | - 38 | 24 | 48 | 3.9 | 379 |
| Right Superior Frontal Gyrus | 8 | 48 | 30 | 3.44 | 329 |
|  | | | | | |
| **Children > Adults** | | | | | |
|  | | | | | |
| Left Lateral Occipital Cortex | - 32 | - 88 | 6 | 4.81 | 4474 |
| Left Hippocampus, Posterior Parahippocampal Gyrus | - 30 | - 30 | - 6 | 4.09 |  |
| Right Lateral Occipital Cortex, Occipital Fusiform Gyrus, Lingual Gyrus | 30 | - 86 | 4 | 4.73 | 1717 |
|  |  |  |  |  |  |
| **Increase Over Time** | | | | | |
|  | | | | | |
| **Young Adults > Children** | | | | | |
|  | | | | | |
| Left Lateral Occipital Cortex | - 32 | - 88 | 6 | 4.81 | 4474 |
| Left Hippocampus | - 30 | - 30 | - 6 | 4.09 |  |
| Left Lingual gyrus | - 10 | - 56 | - 6 | 4.04 |  |
| Right Lateral Occipital Cortex, Occipital Fusiform Gyrus, Precuneus | - 30 | 86 | 4 | 4.73 | 1717 |
|  |  |  |  |  |  |
| **Children > Young Adults** | | | | | |
|  |  |  |  |  |  |
| Right Superior Parietal Lobule, Angular Gyrus | 42 | - 50 | 58 | 3.69 | 946 |
| Right Middle Frontal Gyrus | 42 | 56 | 2 | 4.16 | 546 |
| Left Middle Frontal Gyrus, Superior Frontal Gyrus | - 38 | 24 | 48 | 3.9 | 379 |
| Right Superior Frontal Gyrus, Paracingulate Gyrus | 8 | 48 | 30 | 3.44 | 329 |

*Regions exhibiting stronger activation for remote vs. recent items in (i) young adults, (ii) children, (iii) children vs young adults, and (iv) young adults vs children on Day 1 (short delay). To capture the involved brain region better, local maxima are presented in addition to cluster maxima for the largest clusters. The preprocessing steps did not include global signal regression.*

| **Day 1 (Short Delay)** | | | | |  |
| --- | --- | --- | --- | --- | --- |
| **(i)Young adults** | | | | | |
| **Region** | **x** | **y** | **x** | **Z-max** | **# voxels** |
|  |  |  |  |  |  |
| Left Lateral Occipital Cortex | - 32 | -78 | 32 | 6.07 | 1598 |
| Left Inferior Temporal Gyrus, Occipital Fusiform Gyrus | - 44 | - 60 | -12 | 6.01 | 10855 |
| Left Posterior Temporal Fusiform Gyrus | - 30 | - 42 | -14 | 4.94 |  |
| Left Cerebral White Matter | - 34 | - 54 | -4 | 4.46 | 1661 |
| Left Parahippocampal Gyrus, Posterior | - 36 | - 22 | -26 | 4.42 |  |
| Precentral Gyrus, Middle Frontal Gyrus | -44 | 0 | 40 | 6.13 | 851 |
| Inferior Frontal Gyrus, Pars Opercularis | -50 | 10 | 18 | 3.98 |  |
| Right Lateral Occipital Cortex | 28 | -68 | 48 | 5.19 | 533 |
| Left Superior/Middle Frontal Gyrus | -24 | 0 | 50 | 5.6 | 345 |
| Left Inferior Frontal Gyrus, Pars Triangularis | -54 | 34 | 4 | 3.8 | 273 |
| Right Posterior Parahippocampal Gyrus, Posterior | 22 | -34 | -12 | 4.47 | 256 |
| Cerebellum | 10 | -76 | -22 | 4.2 | 247 |
| Right Middle Inferior Temporal Gyrus | 52 | -54 | -12 | 4.41 | 195 |
| Right Insular Cortex | 30 | 22 | 2 | 4.98 | 172 |
| Right Middle Frontal Gyrus | 34 | 32 | 16 | 3.36 | 171 |
|  | | | | | |
| **(ii)Children** | | | | | |
|  |  |  |  |  |  |
| Left Temporal Fusiform Cortex, | -36 | -40 | -12 | 4.76 | 513 |
| Left Parahippocampal Gyrus, Posterior | -16 | -42 | -10 | 4.71 |  |
| Right Temporal Fusiform Gyrus | 40 | -30 | -18 | 4.77 | 392 |
| Right Parahippocampal Gyrus, Posterior | 26 | -38 | -10 | 4.36 |  |
|  |  |  |  |  |  |
| **(iii)Children > Young Adults** | | | | | |
|  |  |  |  |  |  |
| Right Pariental Cortex, Precuneus | 4 | -48 | 30 | 5.16 | 1031 |
| Precuneus | -4 | -48 | 38 | 4.23 |  |
|  |  |  |  |  |  |
| **(iv)Young Adults > Children** | | | | | |
|  |  |  |  |  |  |
| Left Middle Frontal Gyrus | -44 | 2 | 40 | 4.02 | 204 |
| Left Inferior Frontal Gyrus, Pars Opercularis | -52 | 8 | 32 | 3.86 |  |
| Left Frontal Orbital Cortex, Operculum Cortex | -38 | 22 | -4 | 4.04 | 151 |
| Left Frontal Pole | -48 | 44 | 4 | 4.08 | 127 |

*Regions exhibiting stronger activation for remote vs. recent items in (i) young adults, (ii) children, (iii) children vs young adults, and (iv) young adults vs children on Day 14 (long delay). To capture the involved brain region better, local maxima are presented in addition to cluster maxima for the largest clusters. The preprocessing steps did not include global signal regression.*

| **Day 14 (Long Delay)** | | | | | |
| --- | --- | --- | --- | --- | --- |
| **(i)Young Adults** | | | | | |
| **Region** | **x** | **y** | **x** | **Z-max** | **# voxels** |
| Right Inferior Temporal Gyrus | 50 | -58 | -14 | 7.6 | 22203 |
| Left Lateral Occipital Cortex | -34 | -84 | 22 | 7.47 |  |
| Right Temporal Fusiform Cortex | 32 | -38 | -18 | 7.47 |  |
| Left Inferior Frontal Gyrus, Pars Opercularis | -42 | 12 | 28 | 7.1 | 2319 |
| Middle Frontal Gyrus | -50 | 22 | 30 | 6.67 |  |
| Right Inferior Frontal Gyrus | 42 | 28 | 22 | 4.54 | 1084 |
| Right Middle Frontal Gyrus | 44 | 32 | 20 | 4.48 |  |
| Caudate | -12 | 12 | 4 | 5.24 | 1101 |
| Left Frontal Orbital Cortex | -36 | 34 | -10 | 5.09 | 517 |
| Cerebellum | 22 | -34 | -42 | 6.95 | 369 |
|  | | | | | |
| **(ii)Children** | | | | | |
| Left Temporal Fusiform Cortex | -34 | -26 | -24 | 4.78 | 541 |
| Left Parahippocampal Gyrus, Anterior Devision | -36 | -16 | -24 | 4.41 |  |
| Left Lateral Occipital Cortex | 50 | -70 | -10 | 4.64 | 509 |
|  |  |  |  |  |  |
| **(iii)Children > Young Adults** | | | | | |
|  |  |  |  |  |  |
| Right Angular Gyrus, Parietal Lobe | 60 | -52 | 38 | 4.93 | 672 |
| Right Lateral Occipital Cortex | 46 | -66 | 48 | 4.4 |  |
| Right Cingulate Gyrus | 8 | -50 | 30 | 4.41 | 310 |
| Right Precuneus Cortex | 10 | -54 | 38 | 4.36 |  |
| Right Parietal Operculum Cortex | 54 | -26 | 22 | 3.87 | 241 |
| Right Frontal Medial Cortex | 10 | 50 | -2 | 4.32 | 141 |
| Left Precuneus Cortex | -6 | -50 | 40 | 4.45 | 131 |
| Left Insula Cortex | -32 | 8 | 12 | 3.97 | 109 |
|  |  |  |  |  |  |
| **(iv)Young Adults > Children** | | | | | |
|  |  |  |  |  |  |
| Right Cerebellum | 14 | -72 | -24 | 5.05 | 1471 |
| Left cerebellum | -30 | -80 | 36 | 5.1 | 514 |
| Left Lateral Occipital Cortex | -34 | -84 | 22 | 3.65 |  |
| Left Occipital Pole | -18 | -94 | -14 | 4.4 | 492 |
| Right Lateral Occipital Cortex | 36 | -76 | 34 | 4.57 | 366 |
| Left Middle Frontal Gyrus | -44 | 12 | 30 | 4.36 | 235 |
| Cerebellum | -32 | -68 | -54 | 5.25 | 177 |
| Left Precuneus Cortex | -16 | -62 | 18 | 4.86 | 108 |

*Regions exhibiting stronger activation for remote vs. recent items that decreases over time (i) in young adults stronger than in children, (ii) children stronger than in adults, in (iii) young adult and (iv) children; that increases over time (iii) in young adults stronger than in children, and (iv) in children stronger than in young adults, in (iii) young adult and (iv) children. To capture the involved brain region better, local maxima are presented in addition to cluster maxima for the largest clusters. The preprocessing steps did not include global signal regression.*

| **Decrease Across Time** | | | | | |
| --- | --- | --- | --- | --- | --- |
|  | | | | | |
| **(ii)Children > Adults** | | | | | |
|  | | | | | |
| **Region** | **x** | **y** | **x** | **Z-max** | **# voxels** |
| Right Occipital Pole | 18 | -92 | 2 | 4.48 | 1195 |
| Left Lateral Occipital Cortex | -32 | -88 | 6 | 4.39 | 1141 |
| Right Precuneus Cortex | 10 | -52 | 10 | 3.66 | 588 |
|  |  |  |  |  |  |
| **(iii)Adults** | | | | | |
| Right Angular Gyrus | 60 | -50 | 40 | 4.02 | 1533 |
| Right Lateral Occipital Cortex | 52 | -58 | 46 | 3.5 |  |
| Right Middle Temporal Gyrus | 70 | -22 | -18 | 4.39 | 641 |
| Right Frontal Pole | 44 | 56 | -6 | 4.89 | 543 |
| Left Lateral Occipital Cortex | -46 | -62 | 38 | 4.1 | 378 |
|  | | | | | |
| **Increase Over Time** | | | | | |
|  | | | | | |
| **(i)Young Adults > Children** | | | | | |
|  | | | | | |
| Right Occipital Pole | 18 | -92 | -14 | 4.48 | 1195 |
| Left Lateral Occipital Cortex | -32 | -88 | 6 | 4.39 | 1141 |
| Right Precuneus Cortex | 10 | -52 | 10 | 3.66 | 588 |
|  |  |  |  |  |  |
| **(iii)Young Adults** | | | | | |
| Left Lateral Occipital Cortex | -42 | -84 | -10 | 6.17 | 16779 |
| Right Lateral Occipital Cortex | 36 | -88 | 8 | 5.97 |  |
| Left Caudate, Thalamus, |  |  |  | 4.25 | 1455 |
| Lfer Insular Cortex, Inferior Frontal Gyrus, Triangularis, Frontal Orbital Cortex | -30 | 28 | 6 | 3.95 |  |
| Left Middle Frontal Gyrus | -38 | 18 | 32 | 4.67 | 759 |
| Left Inferior Frontal Gyrus, Pars Opercularis | -44 | 14 | 28 | 4.44 |  |
| Right Inferior Frontal Gyrus, Pars Opercularis, Pars Triangularis, | 46 | 12 | 28 | 3.6 | 468 |
| Right Middle Frontal Gyrus | 46 | 24 | 28 | 3.13 |  |
|  |  |  |  |  |  |
| **(iv)Children** | | | | | |
| Left Insular Cortex, Frontal Operculum Cortex | -32 | 12 | 12 | 4.14 | 1262 |
| Left Frontal Orbital Cortex | -40 | 22 | -4 | 3.54 |  |
| Left Inferior Frontal Gyrus, Pars Opercularis | -32 | 22 | 22 | 3.47 |  |
