# Supplementary material for: Neural correlates and reinstatement of recent and remote memory in children and young adults
Source: eLife. 2025 Dec 5;12:RP89908. doi: 10.7554/eLife.89908 (PMC12680376; doi:10.7554/eLife.89908)
Supplement: Supplementary file 8. [file elife-89908-supp8.docx]

Supplementary File 8

*Full statistical overview of LME model for univariate analysis (sub-sampled to those participants who reached accuracy criteria after 2 encoding loops).*

|  | **Main Effect**  **of Group** | | | **Main Effect**  **of Session** | | **Group x Session Interaction** | |  |
| --- | --- | --- | --- | --- | --- | --- | --- | --- |
| ***Regions of Interest*** | *F_(DF)_* | | *p* | *F_(DF)_* | *p* | *F_(DF)_* | p | *R2* |
| Hippocampus Anterior | .02_(1,125)_ | .894 | | .931_(1,125)_ | .336_(.707)_ | .701_(1,125)_ | .404_(.494)_ | .042⊥ |
| Hippocampus Posterior | 1.62_(1,125)_ | | .206_(_ | 3.51_(1,125)_ | .063_(.939)_ | 1.73_(1,125)_ | .191_(.607)_ | .141⊥ |
| Parahippocampal Gyrus Anterior | .147_(1,125)_ | | .702 | .496_(1,125)_ | .482_(.707)_ | .110_(1,125)_ | .740_(.680)_ | .051∩ |
| Parahippocampal Gyrus Posterior | 5.22_(1,61)_ | | .021 | .440_(1,71)_ | .509_(.330)_ | 22.05_(1,58)_ | <.001_(.095)_ | .482⊥ |
| Medial Prefrontal Cortex | 11.25_(1,66)_ | | .0013_(.293)_ | .036_(1,75)_ | .851_.568)_ | .224_(1,69)_ | .638_(.953)_ | .468⊥ |
| Ventrolateral Prefrontal Cortex | 14.93_(1,65)_ | | **<.001_(<.001)_** | 9.91_(1,79)_ | **.002_(.035)_** | 1.48_(1,65)_ | .228_(.494)_ | .313⊥ |
| Cerebellum | 5.60_(1,125)_ | | .019_(.816)_ | 1.09_(1,125)_ | .299_(.607)_ | 15.03_(1,125)_ | <.001_(.060)_ | .204∩ |
| Retrosplenial Cortex | .01_(1,125)_ | | .936_(.877)_ | 7.06_(1,125)_ | .009_(.568)_ | 14.40_(1,137)_ | <.001_(.060)_ | .150∩ |
| Precuneus | 4.51_(1,65)_ | | .038_(.790)_ | 13.41_(1,79)_ | .0005_(.330)_ | 5.60_(1,65)_ | .021_(.194)_ | .171∩ |
| Lateral Occipital Cortex | 8.81_(1,64)_ | | .004_(.095)_ | 16.04_(1,77)_ | **.0001_(.02)_** | 4.73_(1,64)_ | .033_(.060)_ | .290⊥ |

*Notes.* *Notes.* Subject was included as random effect. Group (children, young adults), Session (Day 1 remote > recent, Day 14 remote > recent), and their interaction were included as fixed effect. The following reference levels where used: for Session – Day 1; for Group – Children; F – F-value; DF – degrees of freedom; p – p-value; FDR_adj – False Discovery Rate adjusted; R2 – amount of variance explained by the model (∩- marginal; ⊥ - conditional). Type III Analysis of Variance Table with Satterthwaite’s method. *p < .05; ** < .01, *** < .001 (significant difference). All p-values of main and interactions effects were FDR-adjusted for multiple comparisons.
