# Supplementary material for: Neural correlates and reinstatement of recent and remote memory in children and young adults
Source: eLife. 2025 Dec 5;12:RP89908. doi: 10.7554/eLife.89908 (PMC12680376; doi:10.7554/eLife.89908)
Supplement: Supplementary file 9. [file elife-89908-supp9.docx]

Supplementary File 9

*Statistical overview of the main and interaction effects of the linear mixed effects model for scene-specific reinstatement.*

|  | **Main Effect**  **of Group** | | **Main Effect**  **of Session** | | **Group x Session Interaction** | | **Main Effect**  **of BOLD activation** | |  |
| --- | --- | --- | --- | --- | --- | --- | --- | --- | --- |
| ***Regions of Interest*** | *F_(DF)_* | *p* | *F_(DF)_* | *p* | *F_(DF)_* | *p* | *F_(DF)_* | *p* | *R2* |
| HCa | 27.21_(1,86)_ | **<.001** | 100.70_(2,159)_ | **<.001** | .94_(2,159)_ | .393 | .92_(1,226)_ | .339 | .411 |
| HCp | 27.19_(1,87)_ | **<.001** | 98.18_(2,159)_ | **<.001** | 1.71(_2,158)_ | .183 | .97_(1,240)_ | .324 | .417 |
| PHGa | 23.14_(1,87)_ | **<.001** | 97.74_(2,159)_ | **<.001** | 1.62_(2,159)_ | .201 | 1.05_(1,221)_ | .307 | .397 |
| PHGp | 15.70_(1,82)_ | **<.001** | 94.40_(2,163)_ | **<.001** | 1.85(_2,155)_ | .161 | .25_(1,240)_ | .619 | .371 |
| mPFC | 8.89_(1,90)_ | **.0044** | 72.811_(2,161)_ | **<.001** | .935_(2,152)_ | .395 | 2.24_(1,221)_ | .136 | .634 |
| vlPFC | 15.18_(1,90)_ | **<.001** | 71.36_(2,172)_ | **<.001** | 1.23_(2,165)_ | .295 | .003_(1,242)_ | .955 | .591 |
| CE | 9.54_(1,87)_ | **.0038** | 59.99_(2,166)_ | **<.001** | 1.17_(2,162)_ | .313 | .679_(1,228)_ | .411 | .520 |
| RSC | 9.27_(1,89)_ | **.0038** | 79.40_(2,162)_ | **<.001** | 1.86_(2,162_ | .159 | .101_(1,242)_ | .751 | .564 |
| PC | 11.35_(1,85)_ | **.0016** | 74.33_(2,161)_ | **<.001** | 1.57_(1,160)_ | .190 | .008_(1,223)_ | .925 | .580 |
| LOC | 1.22_(1,100)_ | .271 | 64.96_(2,167)_ | **<.001** | 1.05_(2,162)_ | .350 | 1.33(_1,220)_ | .249 | .523 |

*Notes.* Subject was included as a random effect. Group (children, young adults), Delay ( recent, remote (Day 1), remote (Day 14)), and their interaction were included as fixed effect. The following reference levels where used: for Delay, recent; for Group, Children; F – F-value; DF – degrees of freedom; p – p-value; FDR_adj – False Discovery Rate adjusted; R2 – amount of variance explained by the model (Stoffel et al., 2021); mPFC – medial prefrontal cortex; vlPFC – ventrolateral prefrontal cortex; HCa – anterior hippocampus; HCp – posterior hippocampus; PHGa – anterior parahippocampal cortex; PHGp – posterior parahippocampal cortex; CE – cerebellum; PC – precuneus; RSC – retrosplenial cortex; LOC – lateral occipital cortex.. Type III Analysis of Variance Table with Satterthwaite's method. *p < .05; ** < .01, *** < .001 (significant difference). All main and interactions p-values were FDR-adjusted for multiple comparisons. All main and interactions p-values were FDR-adjusted for multiple comparisons.
