# Supplementary material for: Neural correlates and reinstatement of recent and remote memory in children and young adults
Source: eLife. 2025 Dec 5;12:RP89908. doi: 10.7554/eLife.89908 (PMC12680376; doi:10.7554/eLife.89908)
Supplement: Supplementary file 10. [file elife-89908-supp10.docx]

Supplementary File 10

*Statistical overview of the main and interaction effects of the linear mixed effects model for object-specific reinstatement.*

| **Object-specific Reinstatement** | | |  | |  |  |  |  |
| --- | --- | --- | --- | --- | --- | --- | --- | --- |
| *Predictors* | *Estimate* | *CI* | *p-value* | |  |  |  |  |
| (Intercept) | .47  .03  .00  -.01  .00  .00  -.01  .02 | .45 – .48 | **<0.001** | |  |  |  |  |
| Group (adults vs children) |  | .01 – .05 | .013 | |  |  |  |  |
| Session (Day 1 vs Day 14) |  | -.02 – .02 | .765 | |  |  |  |  |
| Condition (recent vs remote) |  | -.03 – .01 | .216 | |  |  |  |  |
| Group × Session |  | **-.03 – .03** | **.927** | |  |  |  |  |
| Group × Condition |  | -.02 – .03 | .817 | |  |  |  |  |
| Session × Condition |  | -.04 – .01 | .343 | |  |  |  |  |
| Group ×Session × Condition |  | -.02 – .06 | .350 | |  |  |  |  |
| **Random Effects** |  | | |  | | | |  |
| σ^2^ | .00 | | |  | |  |  |  |
| τ_00_ _subNo_ | .00 | | |  | |  |  |  |
| ICC | .16 | | |  | |  |  |  |
| N _subNo_ | 83 | | |  | |  |  |  |
| Observations | 3058 | | |  | |  |  |  |
| Marginal R^2^ / Conditional R^2^ | .401/ .498 | | |  | |  |  |  |

*Notes.* CI – confidence interval; p – p-value; σ2 – residuals, τ00 – variance of the random intercept. Type III Analysis of Variance Table with Satterthwaite's method. *p < .05; ** < .01, *** < .001 (significant difference). The output table has been shortened for clarity; all interaction effects, including those involving ROI, were excluded as they did not yield significant results.
