# Supplementary material for: Neural correlates and reinstatement of recent and remote memory in children and young adults
Source: eLife. 2025 Dec 5;12:RP89908. doi: 10.7554/eLife.89908 (PMC12680376; doi:10.7554/eLife.89908)
Supplement: Supplementary file 11. [file elife-89908-supp11.docx]

Supplementary File 11

*Statistical overview of the main and interaction effects of the linear mixed effects model for remote object-specific reinstatement.*

|  | **Main Effect**  **of Session** | | **Group x Session Interaction** | |
| --- | --- | --- | --- | --- |
| ***Regions of Interest*** | *F_(DF)_* | *p* | *F_(DF)_* | *p* |
| HCa | .44_(1,77)_ | .506 | .001_(1,77)_ | .973 |
| HCp | 1.09_(1,77)_ | .299 | .56(_1,77)_ | .456 |
| PHGa | .48_(1,83)_ | .487 | .946_(1,80)_ | .333 |
| PHGp | .395_(1,78)_ | .532 | .486(_1,77)_ | .488 |
| mPFC | 1.225_(1,79)_ | .272 | 1.501_(1,78)_ | .224 |
| vlPFC | 1.058_(1,82)_ | .307 | 2.011_(1,82)_ | .160 |
| CE | 424_(1,153)_ | .516 | 6.03_(1,53)_ | .015 |
| RSC | 2.106_(1,153)_ | .149 | 1.09_(1,153)_ | .289 |
| PC | 3.60_(1,153)_ | .059 | .133_(1,153)_ | .716 |
| LOC | 2.006_(1,74)_ | .161 | 1.074_(1,74)_ | .304 |

*Notes.* Subject was included as a random effect. F – F-value; DF – degrees of freedom; p – p-value; mPFC – medial prefrontal cortex; vlPFC – ventrolateral prefrontal cortex; HCa – anterior hippocampus; HCp – posterior hippocampus; PHGa – anterior parahippocampal cortex; PHGp – posterior parahippocampal cortex; CE – cerebellum; PC – precuneus; RSC – retrosplenial cortex; LOC – lateral occipital cortex. Type III Analysis of Variance Table with Satterthwaite's method. *p < .05; ** < .01, *** < .001 (significant difference).
