# Supplementary material for: Neural correlates and reinstatement of recent and remote memory in children and young adults
Source: eLife. 2025 Dec 5;12:RP89908. doi: 10.7554/eLife.89908 (PMC12680376; doi:10.7554/eLife.89908)
Supplement: Supplementary file 12. [file elife-89908-supp12.docx]

Supplementary File 12

*Statistical overview of the main and interaction effects of the linear mixed effects model for scene-specific reinstatement for corpus callosum subregions.*

|  | ***Session*** | | ***Group*** | |
| --- | --- | --- | --- | --- |
| ***ROI*** | ***F_(DF)_*** | ***p*** | ***F_(DF)_*** | ***p*** |
| Corpus Collosum – corpus | 3.88_(2,367)_ | **.021** | 1.33_(1,367)_ | .248 |
| Corpus Collosum – gernu | 3.87_(2,373)_ | **.022** | 1.69_(1,373)_ | .195 |
| Corpus Collosum – splenium | 3.88_(2,373)_ | **.022** | .405_(1,373)_ | .525 |

*Notes.* Subject was included as a random effect. Group (children, young adults), Session ( recent, remote (Day 1), remote (Day 14)) as fixed effect. The following reference levels where used: for Session, recent; for Group, Children; F – F-value; DF – degrees of freedom; p – p-value; Type III Analysis of Variance Table with Satterthwaite's method. *p < .05; ** < .01, *** < .001 (significant difference).
