# Supplementary material for: Neural correlates and reinstatement of recent and remote memory in children and young adults
Source: eLife. 2025 Dec 5;12:RP89908. doi: 10.7554/eLife.89908 (PMC12680376; doi:10.7554/eLife.89908)
Supplement: Supplementary file 13. [file elife-89908-supp13.docx]

Supplementary File 13

*Statistical overview of LME-model based Sidak corrected post hoc comparisons for scene-specific reinstatement analysis for corpus callosum subregions (based on LME-model described in Table S10.3).*

|  | Recent > Remote Day1 | | | Remote Day 1 > Day 14 | | |
| --- | --- | --- | --- | --- | --- | --- |
|  | *b* | *t_(DF)_* | *p* | *b* | *t_(DF)_* | *p* |
| Corpus Collosum-Corpus | .029 | 2.23_(156)_ | .080 | .024 | 1.78_(163)_ | .213 |
| Corpus Collosum - gernu | .027 | 2.05_(159)_ | .121 | .028 | 2.04_(165)_ | .124 |
| Corpus Collosum- splenium | .026 | 2.03_(159)_ | .128 | .026 | 1.94_(166)_ | .147 |

*Notes.* Degrees of freedom were adjusted based on Kenward-Roger methods. b – Beta values; t – t-value; DF – degrees of freedom; p – p-value; *p < .05; ** < .01, *** < .001 (significant difference).
