# Supplementary material for: Neural correlates and reinstatement of recent and remote memory in children and young adults
Source: eLife. 2025 Dec 5;12:RP89908. doi: 10.7554/eLife.89908 (PMC12680376; doi:10.7554/eLife.89908)
Supplement: Supplementary file 14. [file elife-89908-supp14.docx]

Supplementary File 14

*Test of gist-like representations index for significance (higher than zero).*

|  |  | Recent Pre-activation | | | Short-Delay Pre-activation | | | | | | Long-Delay Pre-activation | | | | | | | | |  |
| --- | --- | --- | --- | --- | --- | --- | --- | --- | --- | --- | --- | --- | --- | --- | --- | --- | --- | --- | --- | --- |
|  |  |  |  |  |  |  |  |  |  |  |  |  |  |  |  |  |  |  |  |  |
|  |  | **Children** | | | | | | | | | | | | | | | | | |  |
| **ROI** | *mean* | | *p* | *p_(FDRadj)_* | | *mean* | *p* | | | *p_(FDRadj)_* | | | | *mean* | | *p* | | *p _(FDRadj)_* |  |  |
| mPFC | .006 | | .135 | .303 | | .003 | .282 | | | .346 | | | **.026** | | | **.002** | | **.013** |  |  |
| vlPFC | -.001 | | .583 | .583 | | .006 | .088 | | | .178 | | | **.020** | | | **.001** | | **.007** |  |  |
| HCa | .003 | | .045 | .135 | | .004 | .038 | | | .135 | | | -.001 | | | .586 | | .704 |  |  |
| HCp | .002 | | .076 | .196 | | .003 | .097 | | | .196 | | | .004 | | | .227 | | .340 |  |  |
| PHGa | .002 | | .225 | .415 | | .005 | .018 | | | .113 | | | .002 | | | .328 | | .415 |  |  |
| PHGp | .003 | | .127 | .254 | | .004 | .045 | | | .181 | | | -.001 | | | .617 | | .741 |  |  |
| CE | .001 | | .373 | .590 | | -.001 | .553 | | | .590 | | | .012 | | | .125 | | .590 |  |  |
| PC | .004 | | .066 | .099 | | **.009** | **.007** | | | **.044** | | | .012 | | | .042 | | .085 |  |  |
| RSC | .004 | | .019 | .090 | | .006 | .030 | | | .090 | | | .010 | | | .053 | | .105 |  |  |
| LOC | .002 | | .253 | .304 | | **.011** | **.008** | | | **.024** | | | .009 | | | .107 | | .161 |  |  |
|  |  | **Young Adults** | | | | | | | | | | | | | | | | | |  |
|  | *mean* | | *p* | *p_(FDRadj)_* | *mean* | | | *p* | *p_(FDRadj)_* | | | *mean* | | | *p* | | *p_(FDRadj_* | | | |
| mPFC | .002 | | .151 | .303 | -.00002 | | | .553 | .553 | | | .002 | | | .288 | | .346 | | | |
| vlPFC | .001 | | .357 | .532 | .005 | | | .063 | .178 | | | .001 | | | .443 | | .532 | | | |
| HCa | .002 | | .116 | .232 | .001 | | | .208 | .313 | | | -.002 | | | .895 | | .900 | | | |
| HCp | .0003 | | .379 | .456 | .003 | | | .016 | .096 | | | -.003 | | | .882 | | .883 | | | |
| PHGa | .002 | | .082 | .245 | .001 | | | .346 | .415 | | | -.004 | | | .909 | | .909 | | | |
| PHGp | .001 | | .189 | .285 | .003 | | | .060 | .181 | | | -.004 | | | .939 | | .940 | | | |
| CE | .0002 | | .455 | .590 | .001 | | | .278 | .590 | | | -.0008 | | | .590 | | .590 | | | |
| PC | .002 | | .125 | .150 | .004 | | | .032 | .085 | | | -.001 | | | .567 | | .568 | | | |
| RSC | .002 | | .070 | .106 | .002 | | | .097 | .116 | | | -.001 | | | .678 | | .679 | | | |
| LOC | .004 | | .039 | .078 | **.006** | | | **.004** | **.024** | | | -.002 | | | .779 | | .779 | | | |

*Notes*.To test for significance we used one-sample permutation t-test for more robust calculations with Monte-Carlo permutation percentile confidence interval. The p-values of child group were corrected for False Discovery Rate (FDR) for multiple comparisons. ROI – region of interest; p – p-value; FDRadj – False Discovery Rate adjustment; mPFC – medial prefrontal cortex; vlPFC – ventrolateral prefrontal cortex; HCa – anterior hippocampus; HCp – posterior hippocampus; PHGa – anterior parahippocampal cortex; PHGp – posterior parahippocampal cortex; CE – cerebellum; PC – precuneus; RSC – retrosplenial cortex; LOC – lateral occipital cortex. *p < .05; ** < .01, *** < .001 (significant difference).
