# Supplementary material for: Neural correlates and reinstatement of recent and remote memory in children and young adults
Source: eLife. 2025 Dec 5;12:RP89908. doi: 10.7554/eLife.89908 (PMC12680376; doi:10.7554/eLife.89908)
Supplement: Supplementary file 15. [file elife-89908-supp15.docx]

**Supplementary File 15**

*Memory Strength across Time*

To analyse the time-related change in the memory strength, we employed the drift diffusion modelling approach (Forstmann et al., 2016; Fudenberg et al., 2020; Ratcliff & McKoon, 2008; Wagenmakers et al., 2007a). This approach utilizes performance accuracy and reaction time. We calculated following parameters: (i) the drift rate (v), which indicates memory strength or the average rate of evidence accumulation; (ii) the boundary (a) parameter, which indicates the amount of evidence required to decide or stringency of the decision; (iii) the non-decision time (Ter), which reflects sensorimotor processing time. The analysis was based on the EZ-diffusion model (Wagenmakers et al., 2007). In this model, the parameters are estimated based on memory performance accuracy, the mean and the variance of reaction time of the correct responses. With the derived parameters, we conducted linear mixed-effect models (LME model) for memory measures using the lmer function from the lme4 package in R (Bates et al., 2015) and lmerTest (Kuznetsova et al., 2017). All LME models were calculated with maximum-likelihood estimation and Subject as the random intercept to account for between-subject variability in the derived parameters of the drift diffusion model. For that, we included the within-subject factor of *Session* (Day 0, Day 1, and Day 14) and the between-subject factor of *Group* (children and young adults) in the LME models. All main and interaction effects were False Discovery Rate adjusted for multiple comparisons.

To characterize the change in memory strength across time within and between child and adult groups, we employed the drift diffusion modelling approach (Forstmann et al., 2016; Fudenberg et al., 2020; Ratcliff & McKoon, 2008; Wagenmakers et al., 2007a) that utilizes not only performance accuracy but also reaction time in complex tasks (Criss, 2010; Lerche & Voss, 2019; Palada et al., 2016; Zhou et al., 2021) and can be applied for different developmental groups (Ratcliff et al., 2011, 2012). We calculated (i) the drift rate (v), which indicates the average rate of evidence accumulation in favour of a correct decision. Thus, the drift rate reflects accessibility of memory representations: a higher value indicates a greater probability of making a correct decision, indicating stronger memory. Conversely, lower values suggest slower accumulation of evidence, possibly indicating difficulty in processing information or a lower signal-to-noise ratio strength (Turker & Swallow, 2022). Further, we calculated also (ii) the boundary (a) parameter, which indicates the amount of evidence required to decide. Larger boundary values mean that more information is needed before deciding, leading to more accurate but slower decisions. Conversely, a smaller boundary value suggests that less information is needed, resulting in faster but potentially less accurate decisions. Lastly, (iii) the non-decision time (Ter) was calculated, reflecting the portion of response time that is not related to decision process. A low non-decision time suggests that most of response time is consumed by actual mnemonic decision process rather than peripheral processes. Conversely, a high non-decision time indicates that a large portion of response time is taken up by processes other than mnemonic decision-making.

All these parameters, namely the drift rate, boundary, and non-decision time, were calculated for children and young adults for recent (immediately retrieved), remote Day 1 and remote Day 2 memory items. For recent memory items, we aggregated the drift rates , the boundary, and non-decision time across two sessions, as there were no significant differences between sessions, as indicated by nonsignificant *Session* and *Session x Group* interactions (all p > .13). Additionally, we conducted LME model analyses for each parameter, with *Subject* as a random factor, and *Group* and *Delay* as fixed effects.

Firstly, the Linear Mixed Effects (LME) model for drift rate (v) explained a significant amount of variance R^2^ = .83, 95% CI [.83 - .88]. We observed a significant main effect of *Group*, F_(1,84)_ = 86.56, p < .001_FDR-adjusted_, w^2^ = .44, indicating a lower overall drift rate in children compared to young adults, b = -.06, t_(89)_ = -8.24, p < .001. There was also a significant *Delay* effect, F_(2,156)_ = 215.43, p < .001 _FDR-adjusted_, w^2^ = .73, showing an overall higher drift rate for recent items compared to remote Day 1 items, b = .02, t_(161)_ = 5.54, p < .001, and the drift rate was significantly higher for remote Day 1 compared to remote Day 14, b = .06, t_(165)_ = 14.64, p < .001. Additionally, there was a significant *Group x Delay* interaction, F_(2,156)_ = 28.08, p < .001 _FDR-adjusted_, w^2^ = .25. Sidak-corrected post hoc tests revealed that the slope of decrease of the drift rate from recent to remote Day 1 was more pronounced in young adults compared to children, b = -.03, t_(161)_ = -4.24, p = <.001, and the slope of decrease of the drift rate from remote Day 1 to remote Day 14 was steeper in young adults, b = -.03, t_(165)_ = -3.28, p = .008. The results show overall lower memory strength in children compared to adults, indicating less effective long-term memory consolidation in children compared to young adults already immediately after learning and extending into longer delays. Albeit adults showed higher memory strength during all delays, the decline rate was faster compared to children, indicating with this profound changes in the memory strength of initially strong memories that stronger memories tend to lose more.

Figure

**Delay-related Change in Memory Strength as Indicated by Drift Rate, Boundary and Non-decision Time Change Within and Between Children and Young Adults.** (A) Drift Rate Change reflects the change in the memory strength or efficiency of evidence accumulation (retrieval processes) to choose a correct item location. (B) Boundary Change reflects the delay-related change in the stringency of retrieval-based decision process. (C) Non-decision time change reflects the delay-based change in sensorimotor processing during memory retrieval decision. **p* < .05; ***p* < .01; ****p* < .001(significant difference); non-significant differences were not specifically highlighted. Error bars indicate standard error based on the underlying LME-model.

Secondly, the LME model for the boundary (a) explained a significant amount of variance R2 = .43, 95% CI [.39 - .53]. It revealed a significant main effect of *Delay*, F_(2,159)_ = 11.32, p = <.001_FDR-adjusted_, w^2^ = .11. The overall boundary remained constant for recent to remote Day 1 items, b = -.008, t_(161)_ = -1.24, p = .520, but was significantly higher for remote Day 1 compared to remote Day 14 memories, b = .03, t_(167)_ = 4.57, p = <.001. Neither the *Group* effect nor the *Group x Delay* interaction was significant (all p > .227), indicating that the boundary and its change over time were similar in children and young adults. Overall, these findings indicate a slight decrease in boundary separation from a short to a long remote delay across both age groups. This decrease might suggest that participants are slightly more inclined to make mnemonic decisions with less evidence after two weeks.

Thirdly, the LME model for the non-decision time (Ter) explained a significant amount of variance R2 = .50, 95% CI [.44 - .60]. The LME revealed a significant main effect of *Delay*, F_(1,157)_ = 3.57, p = .030_FDR-adjusted_, w^2^ = .03. Sidak-adjusted post hoc tests revealed overall lower non-decision time for recent items compared to remote Day 14 items, b = -.13, t_(165)_ = -2.63, p = .028. There was no significant main effect of *Group* (p = .293), indicating similar non-decision time between children and adults. In addition, a significant *Group x Delay* interaction was observed, F_(2,157)_ = 4.32, p = .022 _FDR-adjusted_, w^2^ = .04. The Sidak-adjusted post hoc tests showed significantly higher non-decision time for remote Day 14 memories compared to remote Day 1 memories in young adult, b = .22, t_(164)_ = 3.10, p = .013. This delay-related increase in adults was significantly higher compared to children, b = .28, t_(166)_ = 2.83, p = .031. There were no other significant between or within group difference in the non-decision time (all p > .18). Overall, these findings suggest that overall increase in non-decision time over time was driven by the young adult group.

Table

*Statistical overview of the main and interaction effects of the linear mixed effects model for drift diffusion parameters.*

|  | **Main Effect**  **of Group** | | **Main Effect**  **of Delay** | | **Group x Delay Interaction** | |  |
| --- | --- | --- | --- | --- | --- | --- | --- |
| ***Regions of Interest*** | *F_(DF)_* | p | *F_(DF)_* | *p* | *F_(DF)_* | *p* | *R2* |
| V | 69.56(1,84) | <.001 | 215.43(2,156) | <.001 | 28.08(2,156) | <.001 | .829 |
| A | 1.42(1,85) | .293 | 11.32(2,159) | <.001 | 1.50(2,159) | .227 | .429 |
| Ter | 1.12_(1,85)_ | .293 | 3.57_(2,157)_ | .030 | 4.32_(2,157)_ | .022 | .500 |

*Notes.* Subject was included as random effect. Group (children, young adults), Delay ( recent, remote (Day 1), remote (Day 14)), and their interaction were included as fixed effect. The following reference levels where used: for Delay, recent; for Group, Children; V – drift rate; A – boundary; Ter – Non-decision Time; F – F-value; DF – degrees of freedom; p – p-value; R2 – amount of variance explained by the model (Stoffel et al., 2021). All main and interaction effects are False Discovery Rate corrected for multiple comparisons. Type III Analysis of Variance Table with Satterthwaite's method. *p < .05; ** < .01, *** < .001 (significant difference).
