# Supplementary material for: Neural correlates and reinstatement of recent and remote memory in children and young adults
Source: eLife. 2025 Dec 5;12:RP89908. doi: 10.7554/eLife.89908 (PMC12680376; doi:10.7554/eLife.89908)
Supplement: Supplementary file 17. [file elife-89908-supp17.docx]

**Supplementary File 17**

*Statistical overview of the main and interaction effects of the linear mixed effects models* ***for remote > recent*** *univariate results for correctly recognized items (based on the pipeline without global signal).*

|  | **Main Effect**  **of Group** | | **Main Effect**  **of Session** | | **Group x Session Interaction** | |  |
| --- | --- | --- | --- | --- | --- | --- | --- |
| ***Regions of Interest*** | *F_(DF)_* | *p* | *F_(DF)_* | *p* | *F_(DF)_* | p | *R2* |
| Hippocampus Anterior | .002_(1,161)_ | .963_(.956)_ | .081_(1,161)_ | .776_(.862)_ | .034_(1,161)_ | .853_(.930)_ | .01∩ |
| Hippocampus Posterior | .151_(1,161)_ | .698_(.893)_ | .528_(1,161)_ | .468_(.669)_ | .006_(1,161)_ | .930_(.930)_ | .04∩ |
| Parahippocampal Gyrus Anterior | .153_(1,161)_ | .696_(.893)_ | .01_(1,161)_ | .936_(.936)_ | .013_(1,161)_ | .910_(.930)_ | .013∩ |
| Parahippocampal Gyrus Posterior | .898_(1,83)_ | .345_(.690)_ | 3.31_(1,85)_ | .071_(.236)_ | 3.03_(1,85)_ | .085_(.230)_ | .052⊥ |
| Medial Prefrontal Cortex | 2.20_(1,83)_ | .142_(.460)_ | 2.38_(1,85)_ | .125_(.213)_ | .374_(1,85)_ | .542_(.774)_ | .083⊥ |
| Ventrolateral Prefrontal Cortex | 20.37_(1,80)_ | **<.001_(<.001)_** | 8.70_(1,107)_ | **.004_(.02)_** | .921_(1,83)_ | .340_(.675)_ | .269⊥ |
| Cerebellum | 0.08_(1,161)_ | .784_(.893)_ | 1.64_(1,161)_ | .202_(.404)_ | 3.07_(1,161)_ | .082_(.230)_ | .063∩ |
| Retrosplenial Cortex | .06_(1,161)_ | .804_(.893)_ | .084_(1,161)_ | .773_(.862)_ | 2.87_(1,161)_ | .092**_(.230)_** | .036∩ |
| Precuneus | 1.78_(1,161)_ | .184_(.460)_ | .568_(1,161)_ | .452_(.669)_ | .696_(1,161)_ | .405_(.675)_ | .045∩ |
| Lateral Occipital Cortex | 4.53_(1,88)_ | .036_(.180)_ | 13.64_(1,111)_ | **<.001_(<.001)_** | 3.62_(1,88)_ | .060_(.230)_ | .297⊥ |

|  | **Main Effect**  **of Sex** | | **Main Effect**  **of Handedness** | | **Main Effect**  **of IQ** | | **Main Effect**  **Of Reaction Time** | |
| --- | --- | --- | --- | --- | --- | --- | --- | --- |
| ***Regions of Interest*** | *F_(DF)_* | *p* | *F_(DF)_* | *p* | *F_(DF)_* | p | *F_(DF)_* | *p* |
| Hippocampus Anterior | .011_(1,161)_ | .9155 | .681_(1,161)_ | .508 | .036_(1,161)_ | .850 | .293_(1,161)_ | .853 |
| Hippocampus Posterior | .039_(1,161)_ | .843 | 2.73_(1,161)_ | .068 | .002_(1,161)_ | .967 | .01_(1,161)_ | .940 |
| Parahippocampal Gyrus Anterior | .178_(1,161)_ | .674 | .828_(1,161)_ | .439 | .027_(1,161)_ | .870 | .027_(1,161)_ | .870 |
| Parahippocampal Gyrus Posterior | .042_(1,84)_ | .838 | .785_(1,93)_ | .459 | .000_1,84)_ | .987 | .289_(1,155)_ | .598 |
| Medial Prefrontal Cortex | .038_(1,87)_ | .846 | 2.93_(1,95)_ | .058 | 1.71_(1,87)_ | .194 | .090_(1,160)_ | .765 |
| Ventrolateral Prefrontal Cortex | .008_(1,81)_ | .928 | 1.04_(1,90)_ | .357 | .625_(1,81)_ | .431 | .029_(1,149)_ | .864 |
| Cerebellum | 1.22_(1,161)_ | .270 | .537_(1,161)_ | .585 | .0001_(1,161)_ | .991 | 1.07_(1,161)_ | .303 |
| Retrosplenial Cortex | .309_(1,161)_ | .579 | .749_(1,161)_ | .474 | .662_(1,161)_ | .417 | .310_(1,161)_ | .579 |
| Precuneus | .323_(1,161)_ | .570 | .856_(1,161)_ | .427 | .621_(1,161)_ | .432 | .007_(1,161)_ | .934 |
| Lateral Occipital Cortex | .001_(1,88)_ | .977 | 1.13_(1,96)_ | .327 | **7.40_(1,87)_** | **.008** | .000_(1,159)_ | .986 |

*Notes.* *Notes.* Subject was included as random effect. Group (children, young adults), Session (Day 1 remote > recent, Day 14 remote > recent), and their interaction were included as fixed effect. The following reference levels where used: for Session – Day 1; for Group – Children; F – F-value; DF – degrees of freedom; p – p-value; FDR_adj – False Discovery Rate adjusted; R2 – amount of variance explained by the model (∩- marginal; ⊥ - conditional). Type III Analysis of Variance Table with Satterthwaite’s method. *p < .05; ** <.01, ***<.001 (significant difference). All p-values of main and interactions effects were FDR-adjusted for multiple comparisons.

**Mean Signal Differences Between Correct Remote and Recent Memories (without global signal in preprocessing).** The figure presents mean signal difference for remote > recent contrast across sessions and groups during the object presentation time window in **(A)** Anterior and Posterior Hippocampus; (**B**) Anterior and Posterior Parahippocampal Gyrus; **(C)** Cerebellum; **(D)** Medial Prefrontal Cortex; **(E)** Ventrolateral Prefrontal Cortex; **(F)** Precuneus; **(G)** Retrosplenial Cortex; **(H)** Lateral Occipital Cortex. *Note:* Bars indicate the group mean for each session (solid lines for Day 1, dashed lines for Day 14), plotted separately for children and young adults. Error bars represent ±1 standard error of the mean. The colour indicated the age groups: purple for children and khaki yellow for young adults. Across all panels, mean of individual subject data are shown with transparent points. The connecting faint lines reflect within-subject differences across sessions. Orange asterisks denote significant difference of **remote > recent** contrast from zero. An upward orange arrow indicates that this difference is greater than zero, while a downward arrow indicates that this is less than zero. **p* < .05; ***p* < .01; ****p* < .001(significant difference); non-significant differences were not specifically highlighted. Significant main and interaction effects are highlighted by the corresponding asterisks. All main and interaction p-values were FDR-adjusted for multiple comparisons.
