# Supplementary material for: Neural correlates and reinstatement of recent and remote memory in children and young adults
Source: eLife. 2025 Dec 5;12:RP89908. doi: 10.7554/eLife.89908 (PMC12680376; doi:10.7554/eLife.89908)
Supplement: Supplementary file 18. [file elife-89908-supp18.docx]

Supplementary File 18

*Statistical overview of the main and interaction effects of the linear mixed effects model for scene-based univariate neural analysis*

|  | **Main Effect**  **of Group** | | **Main Effect**  **of Delay** | | **Group x Delay Interaction** | |  |
| --- | --- | --- | --- | --- | --- | --- | --- |
| ***Regions of Interest*** | *F_(DF)_* | p | *F_(DF)_* | *p* | *F_(DF)_* | *p* | *R2* |
| HCa | **7.16(1,94)** | **.009** | 2.35(2,238) | .097 | 3.02(2,238) | .051 | .320 |
| HCp | **11.67(1,97)** | **.0009** | **8.25(2,241)** | **.0003** | **7.19(2,241)** | **.0009** | .374 |
| PHGa | **11.02(1,90)** | **.001** | .42(2,234) | .660 | .927(2,234) | .397 | .326 |
| PHGp | .012(1,95) | .914 | **36.46(2,240)** | **<.001** | .749(2,240) | .474 | .377 |
| Medial Prefrontal Cortex | **10.28_(1,85)_** | **.002** | **7.21_(2,163)_** | **<.001** | 2.94 _(2,163)_ | .056 | .105 |
| Ventrolateral Prefrontal Cortex | **5.96_(1,88)_** | **.016** | **55.14_(2,164)_** | **<.001** | **20.47_(2,164)_** | **<.001** | .262 |
| Cerebellum | 1.98_(1,80)_ | .163 | **13.63_(2,158)_** | **<.001** | .065_(2,158)_ | .522 | .084 |
| Retrosplenial Cortex | 1.05_(1,88)_ | .308 | .00_(2,164)_ | .999 | **3.28_(2,164)_** | **.039** | .023 |
| Precuneus | .19_(1,88)_ | .666 | **12.01_(2,163)_** | **<.001** | **4.54_(2,163)_** | **.012** | .056 |
| Lateral Occipital Cortex | **54.52_(1,88)_** | **<.001** | **17.09_(2,163)_** | **<.001** | **3.55_(2,163)_** | **.031** | .338 |

*Notes.* Subject was included as random effect. Group (children, young adults), Delay ( recent, remo te (Day 1), remote (Day 14)), and their interaction were included as fixed effect. The following reference levels where used: for Delay, recent; for Group, Children; mPFC – medial prefrontal cortex; vlPFC – ventrolateral prefrontal cortex; HCa – anterior hippocampus; HCp – posterior hippocampus; PHGa – anterior parahippocampal cortex; PHGp – posterior parahippocampal cortex;CE – cerebellum; PC – precuneus; RSC – retrosplenial cortex; LOC – lateral occipital cortex. F – F-value; DF – degrees of freedom; p – p-value; R2 – amount of variance explained by the model (Stoffel et al., 2021). Type III Analysis of Variance Table with Satterthwaite's method. *p < .05; ** < .01, *** < .001 (significant difference).
